# Evaluation of a novel “gross room” device to increase lymph node yield in colorectal cancer specimens

**DOI:** 10.1016/j.jpi.2026.100745

**Published:** 2026-09-30

**Authors:** Kadir Isidan, Hussein Alnajar, Monika Krezalek, Zitlali Montiel-Martinez, Alicia Huber, John Groth

**Affiliations:** a Department of Pathology and Laboratory Medicine, Endeavor Health, Evanston, IL, USA; b Department of Surgery, Endeavor Health, Evanston, IL, USA

**Keywords:** Cision InVision, Lymph node dissection, Colorectal cancer, Rectal cancer, Gross room

## Abstract

Nodal staging plays a vital role in pathological staging of colorectal cancer (CRC). According to AJCC guidelines, at least 12 lymph nodes are required for nodal staging. However, many previous studies have shown the importance of retrieving as many lymph nodes as possible. In this study, we evaluated an optical imaging-based device (Cision InVision) to increase lymph node yield in CRC specimens. The device utilizes infrared light to differentiate lymphoid tissue from other tissue. We recruited 31 colorectal resections. A senior resident or pathologist's assistant conventionally grossed the recruited specimens. Thereafter, the remaining fatty tissue was scanned with the device to identify additional lymph nodes. Overall, the device increased the lymph node numbers, with an average increase of 48%. This increase was particularly notable for smaller lymph nodes (<3 mm). In the entire study, a total of 1422 lymph nodes were harvested, including 33 positive lymph nodes from 10 nodal-positive cases. Conventional grossing retrieved 29 positive lymph nodes, whereas the device retrieved 4 additional positive nodes, resulting in two upstaged patients. Among these two patients, one patient was upstaged from node-negative to node-positive disease. In this “proof-of-concept” study with a limited number of subjects, our findings demonstrate that the Cision InVision device increases lymph node yield, especially for smaller nodes, leading to stage changes in 2 out of 31 patients, with one case upstaged from node-negative to node-positive disease. Whereas further research with larger cohorts is needed to address the limitations and explore broader clinical implications, our findings preliminarily highlight its probable value in CRC lymph node assessment.

## Introduction

Colorectal cancer (CRC) accounts for 8% (men) and 7% (women) of all annually diagnosed cancers in the United States.^1^ It is the third and fourth most common cause of cancer-related deaths in men and women, respectively.^1^ Surgery remains the main curative treatment approach in CRC.^2^ Depending on tumor characteristics and disease stage, further management may include different chemotherapy regimens and radiotherapy.^3^, ^4^, ^5^

Meticulous pathological examination of CRC specimens is crucial to accurately guide further patient management. Among the quality metrics of pathological examination of colorectal specimens, nodal staging is critical, but it can also be subjective depending on the prosector and the pathologist. American Joint Committee on Cancer guidelines recommend retrieving at least 12 lymph nodes for accurate staging.^6^ However, previous studies showed the importance of retrieving as many lymph nodes as possible, including small lymph nodes.^7^, ^8^, ^9^ The traditional manual lymph node search can be highly subjective, prompting the development of imaging-based tools to improve lymph node retrieval.

Cision InVision (CisionVision, San Mateo, CA) is an optical imaging-based device that utilizes shortwave infrared (SWIR) light to differentiate water-based tissue from lipid-based tissue.^10^ The device is specifically designed for lymph node search and is registered with the Food and Drug Administration (FDA). The device comprises four components: a stage, a screen, two light sources, and a camera (Fig. 1). Two infrared light sources illuminate the stage from inferior-lateral aspects. The camera records the stage from an inferior angle and transmits the image to the screen. Water-based tissue appears black (darker tones), whereas lipid-based tissue appears white-gray (lighter tones). In this way, the device helps the prosector to identify lymph node candidates.

**Fig. 1:**
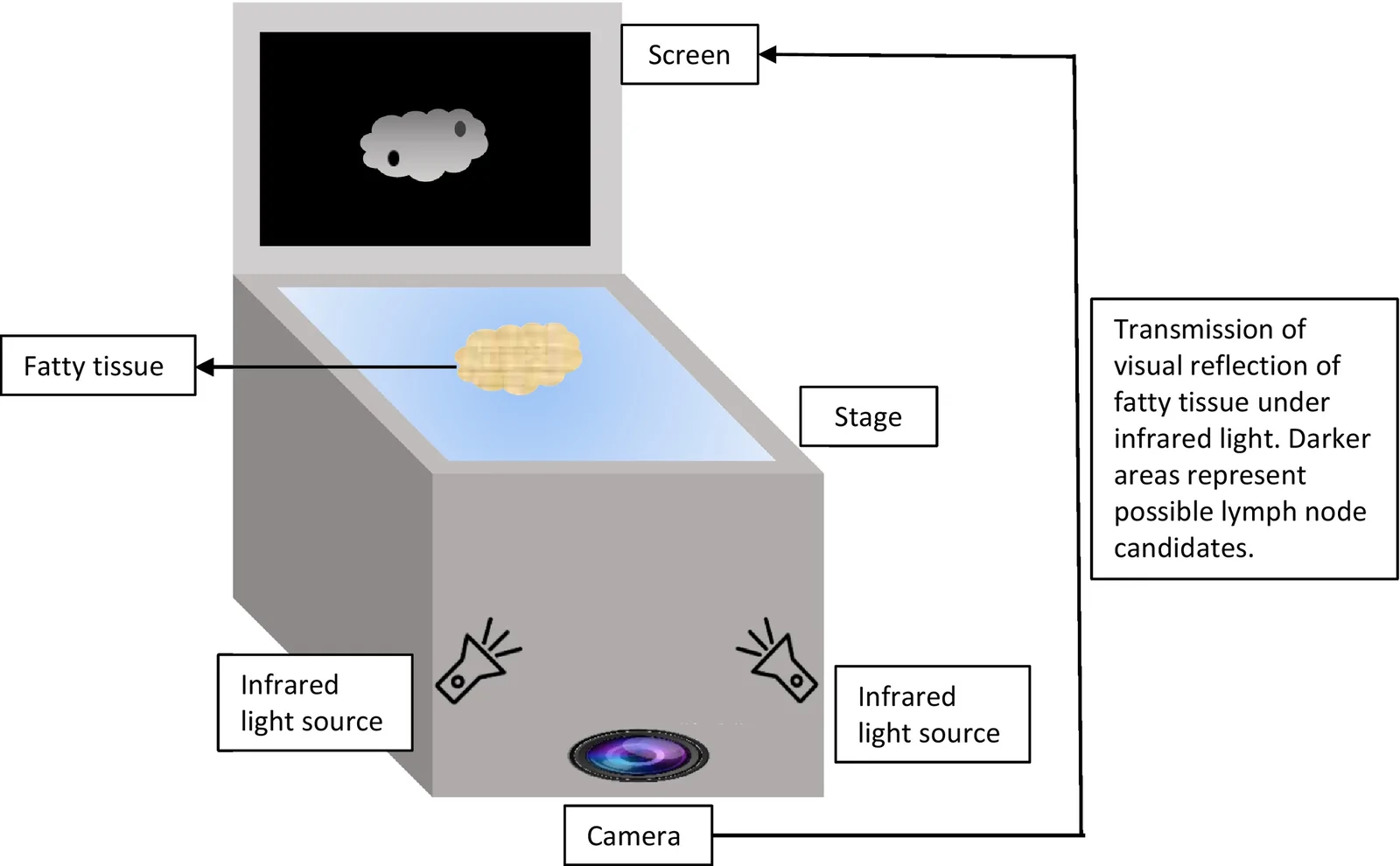
The Cision InVision device with labels for its parts and a summary of its working mechanism.

In this study, we evaluated the Cision InVision device's contribution to lymph node yield in CRC specimens.

## Materials and methods

### Case selection

This study was conducted at NorthShore University (Endeavor Health) hospitals. We considered all colorectal resections with a primary neoplasm as candidate subjects. Thirty-one colorectal resections were recruited to the study (30 colonic adenocarcinoma, 1 sarcomatoid carcinoma) between April and September 2023. The specimens include 11 right hemicolectomies, 9 sigmoidectomies, 7 low-anterior resections (LARs), 2 transverse colectomies, 1 abdomino-perineal resection (APR), and 1 left colectomy. Five LARs, one right hemicolectomy, and one APR were post-neoadjuvant treatment. No other specimens were subjected to neoadjuvant treatment. The inclusion criterion was a specimen measuring 10 cm or greater in its largest dimension. Specimens under 10 cm in their largest dimension and highly fibrotic specimens were excluded (because the device cannot differentiate fibrotic tissue and lymph nodes).

Additionally, we randomly pooled historical controls (*n* = 31) to compare lymph node numbers before and after introducing the Cision InVision device. These cases were from January to December 2022 and matched by tumor site, surgeon, and prosector. Treatment status was matched only in LAR cases. Remaining historical controls were untreated.

### Study procedure

Initially, a senior resident or pathologist's assistant grossed the specimens conventionally. Next, the remaining fatty tissue was scanned with the Cision InVision device to identify additional lymph nodes. All possible lymph node candidates were dissected on the device's stage and retrieved during scanning (Fig. 2). The device's stage allows use of grossing equipment such as blades, scissors, and other dissection instruments. A fat-clearing reagent was available to the prosectors throughout the study. In three random cases (*n* = 3), we submitted the remaining fatty tissue in its entirety after scanning to evaluate whether the device missed any lymph nodes. Finally, an attending pathologist reviewed the slides, and we collected lymph node data (number, size, and positivity). We collected lymph node size data using both gross examination and microscope-stage micrometers.

**Fig. 2:**
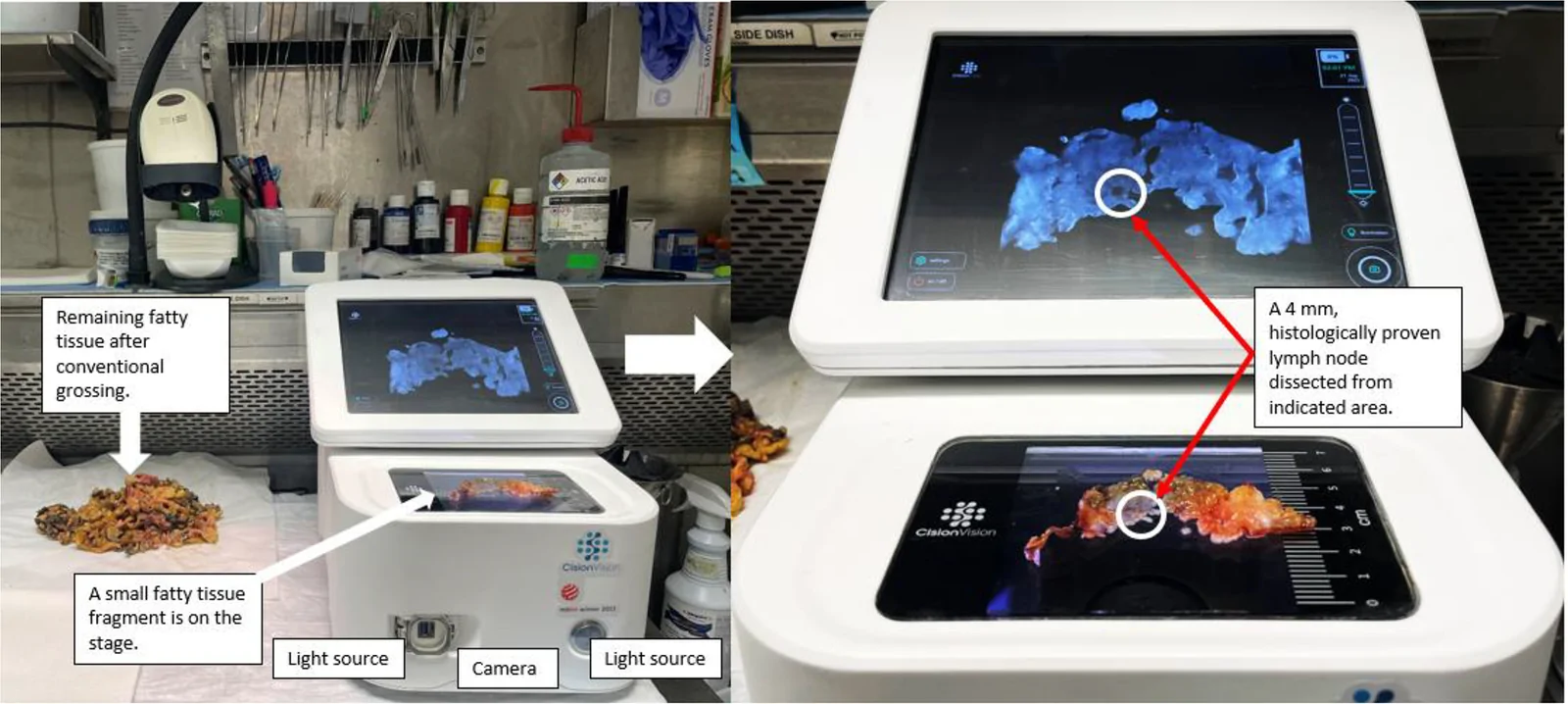
A figure showing the workflow, device parts, and a histologically proven lymph node in fatty tissue. After conventional grossing, the remaining fatty tissue is screened on the stage as thin tissue slices. The image on the right shows a circular darker area on the screen, submitted for histological examination and confirmed as a lymph node.

### Statistical analysis

Wilcoxon signed-rank test with continuity correction (paired) was used to test the null hypothesis that both methods were equal (before vs. after incorporating the Cision InVision device). The significance threshold was set at 0.05, meaning a two-sided α < 0.05 was considered statistically significant in this study.

Mann–Whitney *U* test was used to compare the historical cohort vs. the Cision InVision device. An unpaired *t*-test was used to compare the size difference between lymph nodes retrieved by the prosector and the Cision InVision device. A *p*-value of <0.05 was used as a cutoff for statistical significance in both tests. All statistical analysis was performed using R software (version 4.5.3).

## Results

### Lymph node yield, scanning time, and additional cassettes

The results are summarized in Table 1. The Cision InVision device has increased the lymph node yield (Fig. 3) in all sub-categories as well as when all are combined. The average lymph node numbers in right hemicolectomy specimens increased from 28 to 44 (58%), sigmoidectomy specimens increased from 30 to 44 (46%), and LAR specimens increased from 36 to 53 (46%). The average lymph node count also increased when all subjects were included in the analysis, by 48%.

**Table 1:** Average number of lymph nodes summarized before vs. after device use. The Cision InVision device increased lymph node numbers in all subcategories and in the combined group.

Subcategory	Conventional grossing	Additional lymph nodes per specimen	Conventional grossing + Cision InVision device	*p*-value
Right hemicolectomy (*n* = 11)	28.18 ± 13.0	15.3 ± 9.5	44.7 ± 15.1	*p* < 0.05
Sigmoidectomy (*n* = 9)	30.0 ± 18.8	13.5 ± 5.1	44.0 ± 21.5	*p* < 0.05
Low-anterior resection (*n* = 7)	36.1 ± 17.5	14.4 ± 9.7	53.0 ± 21.3	*p* < 0.05
Others (*n* = 4)	25.5 ± 14.1	14 ± 5.2	40.7 ± 21.2	*p* < 0.05
All combined (*n* = 31)	30.1 ± 16.8	14.5 ± 7.9	45.8 ± 19.8	*p* < 0.001

**Fig. 3:**
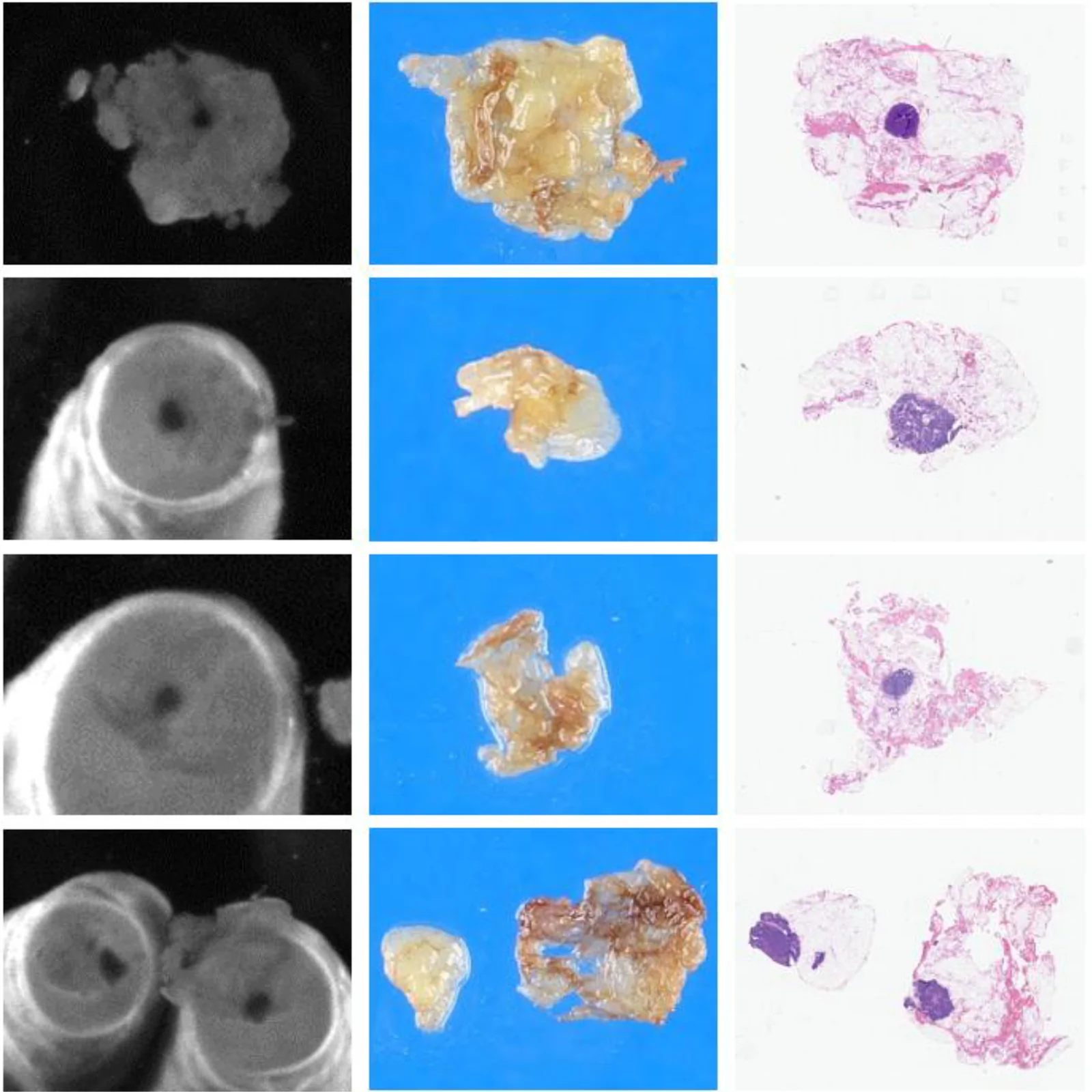
Examples of lymph nodes found by the Cision InVison device. Left column: screen images from the device; middle column: gross images of the same tissue piece; right column: histological images of the same tissue piece.

In three cases, the remaining fatty tissue was entirely submitted to determine whether the device missed any lymph nodes. One case revealed three additional lymph nodes in the remaining fatty tissue (70–73), another revealed one additional lymph node (21–22), and the last also revealed one additional lymph node (59–60). In the three fully submitted cases, the device and prosector combination retrieved 95%–98% of the lymph nodes in the entire fatty tissue, suggesting minimal lymph node loss after optical scanning.

The average lymph node number was found to be 30.0 in the randomly selected case series of historical controls from before the introduction of the Cision InVision device (data not shown). There was no significant difference between the two series (*p* = 0.3; without the device involved). There was an appreciable difference between the historical controls and cases following the introduction of the Cision InVision device (*p* < 0.0001).

The average scanning time was 30 ± 17 min and was directly correlated to the amount of remaining fatty tissue. This was additional time to the routine grossing.

Upon scanning with the Cision InVision device, a total of 131 additional cassettes were submitted for the 31 study cases. The average number of additional cassettes per case was 4.2 ± 2.5. Among the 131 cassettes, 127 (97%) of them showed new lymph nodes, and 4 of them showed only fibroadipose tissue.

### Lymph node size

A total of 1422 lymph nodes were harvested in the entire study. Lymph node size was investigated in eight subgroups, including 0–1 mm (86 nodes), 1–2 mm (317 nodes), 2–3 mm (367 nodes), 3–4 mm (247 nodes), 4–5 mm (128 nodes), 5–7 mm (119 nodes), 7–10 mm (61 nodes), and >10 mm (57 nodes). Fig. 4 shows the relationship between lymph node size and the mean number of lymph nodes per case in that particular size group.

**Fig. 4:**
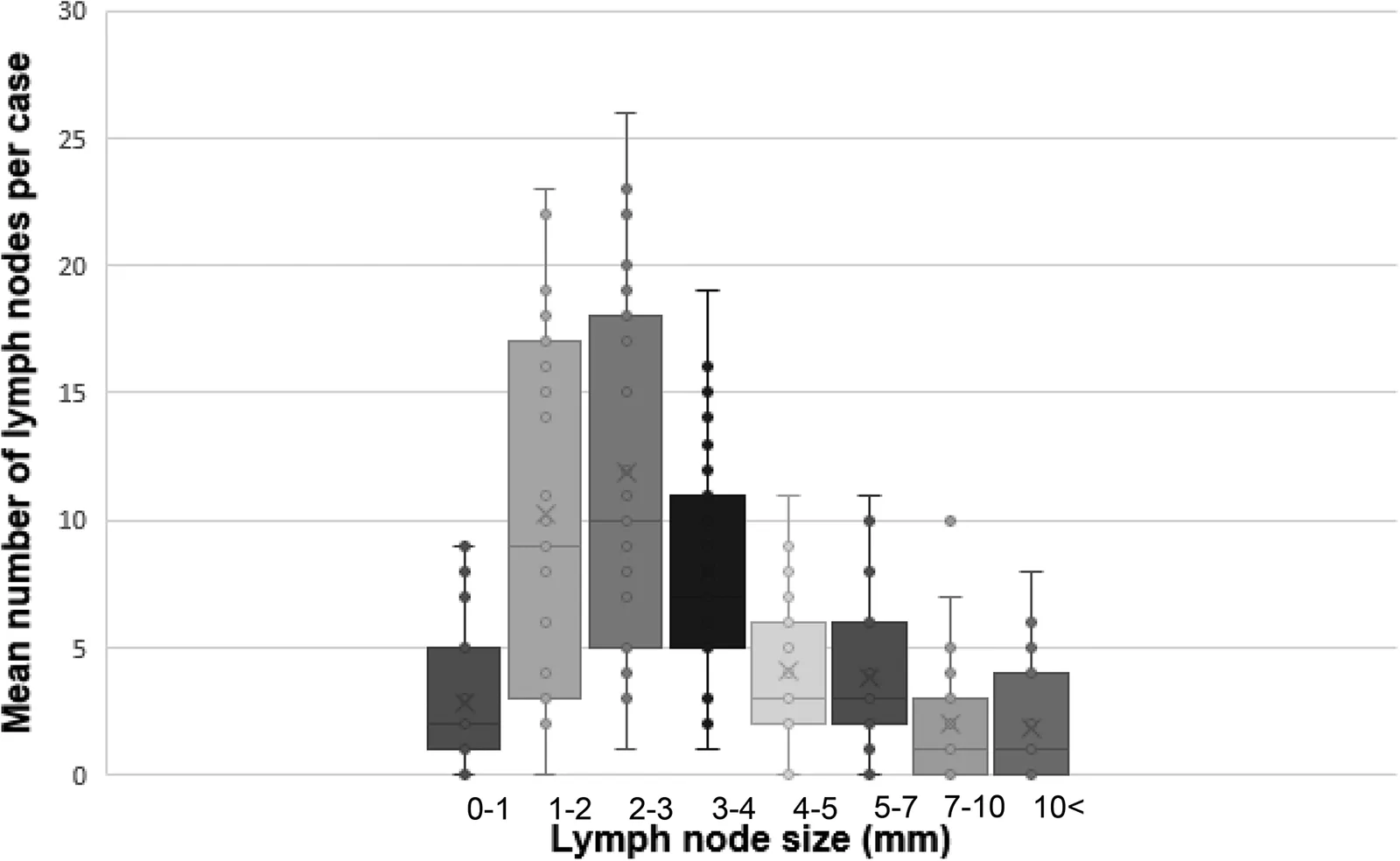
Lymph node size (mm) vs. mean number of lymph nodes per case. This figure shows the distribution of lymph node sizes in colon cases. In our study, the majority of the lymph nodes are found to be between 1 and 4 mm.

### Positive lymph nodes

Among 1422 lymph nodes, 33 positive lymph nodes were identified in 10 nodal-positive cases. Of these, 29 positive lymph nodes were retrieved by conventional grossing and four nodes (in three cases) by the Cision InVision device. In one case, nodal staging changed from +1/13 to +2/26. In the second case, nodal staging changed from +3/74 to +5/89. Finally, in the last case, the nodal staging changed from 0/12 to +1/20, effectively upstaging the patient from node-negative to node-positive. The positive lymph nodes identified by the device measure 2.5, 1.8, 2.2, and 3.3 mm.

No positive lymph node was identified under 1 mm. The size range of positive lymph nodes was as follows: two nodes (1–2 mm), five nodes (2–3 mm), four nodes (3–4 mm), four nodes (4–5 mm), six nodes (5–7 mm), two nodes (7–10 mm), and 10 nodes (>10 mm). Fig. 5 summarizes the proportion of positive lymph nodes in each size group.

**Fig. 5:**
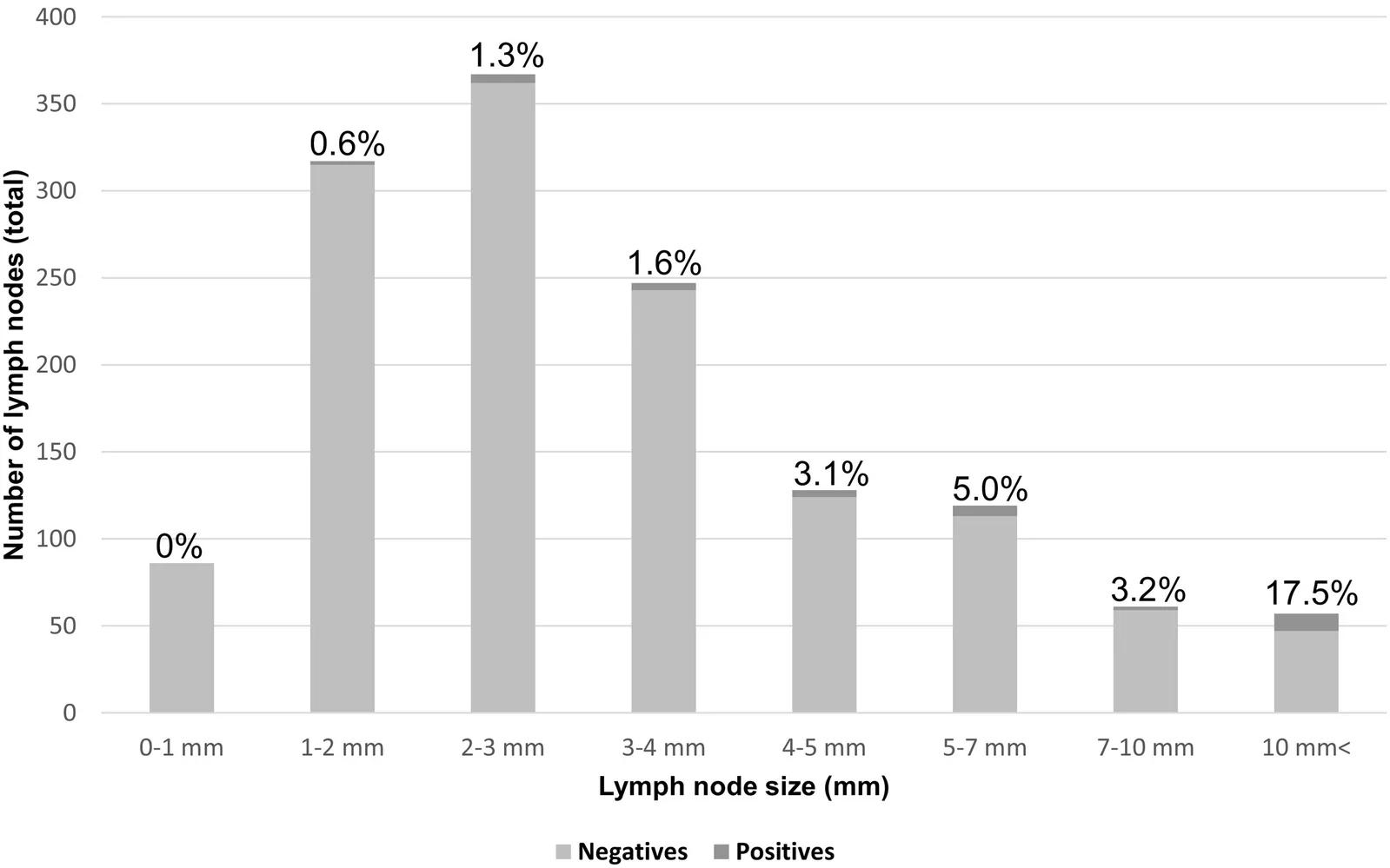
Proportion of positive lymph nodes by size. As expected, very large (>1 cm) lymph nodes have the highest likelihood of being positive by a wide margin, and overall, larger lymph nodes have a higher likelihood of being positive. However, lymph nodes can still be positive even if they are under 2 mm. In our study, we did not find any positive lymph nodes measuring less than 1 mm.

## Discussion

Complete surgical resection remains the optimal treatment approach in CRC.^2^ After surgery, pathological staging and disease features shape the treatment plan and aid prognostication. Among pathological features, lymph node stage, along with T stage, is one of the most significant prognostic factors in CRC. It also plays a vital role in therapeutic decisions. Several variables may affect lymph node yield (and thus nodal stage), including the patient, the surgeon, the prosector, and the pathologist.^11^ Undoubtedly, the quality of the gross examination is the most important modifiable process.

Over the years, manual lymph node search has been the principal mesocolic lymph node search method. Manual search essentially relies on dissection, palpation, and visualization; therefore, it can be highly subjective and depend on experience. Moreover, there is a significant difference in lymph node evaluation between different institutions, leading to possible staging discrepancies and eventually changes in treatment choices and prognosis.^4^, ^5^ Many fat-clearing solutions have been proposed to facilitate the lymph node search, including GEWF solution, Schwartz solution, sodium hypochlorite, glacial acetic acid, diethyl ether, ethanol, etc. All these reagents have been shown to increase the lymph node yield by making the lymph nodes more perceivable and decreasing the density of surrounding fatty tissue.^12^, ^13^, ^14^, ^15^ However, the prosector still relies on the aforementioned basic senses/tools even with these reagents. In our study, although a fat-clearing reagent was available at all times, however, it was not used in any case, based entirely on the prosector's preference. The lack of use of a previously proven lymph node yield-enhancing method is a limitation in our study, especially given the cost difference between the device and fat-clearing reagents.

The main advantage of the Cision InVision device is that it provides the prosector with a new dimension beyond the basic senses: optical imaging. The device uses the natural compositional difference between fatty tissue and lymph nodes to create high-contrast images and display them on a screen in real time. The imaging reflects tissue composition up to a depth of 5 mm. Exposing both sides to the stage with slight top pressure would help better characterize the fatty tissue composition. Due to the shallow penetration, the device might not be as efficient in thick (thicker than 1 cm) tissue pieces, especially for finding smaller lymph nodes. In our experience, the most common false-positive signal (darker areas on the screen) was blood vessels and fibrous tissue (Fig. 6). This problem can usually be solved by manipulating the tissue and viewing it from different angles. Importantly, the Cision InVision device is intended to complement, rather than replace, conventional manual lymph node dissection.

**Fig. 6:**
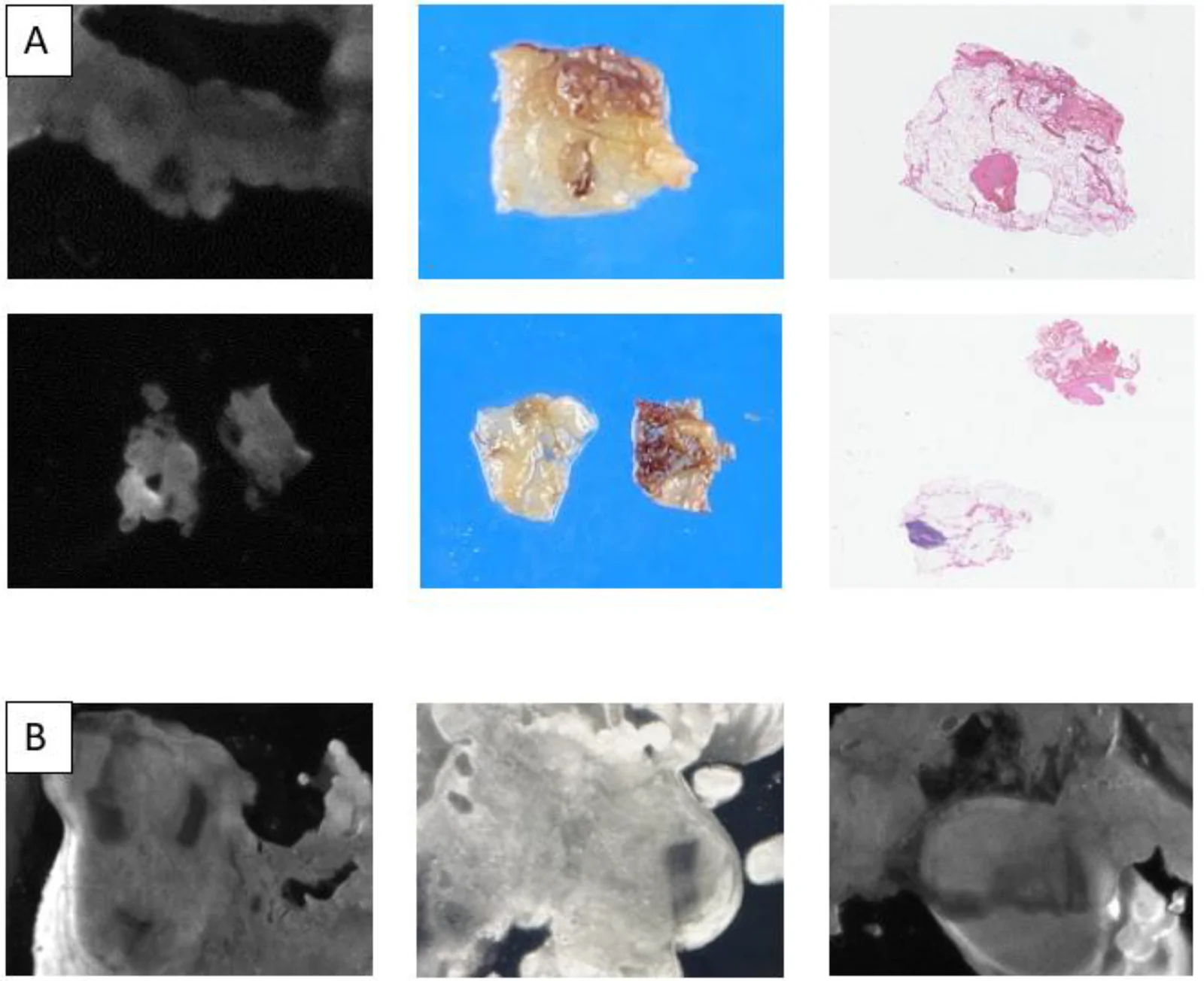
A: Examples of fibrous areas found by the Cision InVison device (the second row also has one lymph node). Left column: screen images from the device; middle column: gross images of the same tissue piece; right column: histological images of the same tissue piece. B: Examples of visualizations of the blood vessels.

The fragmentation of lymph nodes may be a natural result of thorough lymph node dissection and could affect the lymph node count. In our study, the majority of the lymph nodes we encountered were intact. However, we encountered undeniably fragmented lymph nodes in both groups (conventional grossing and Cision InVision) (Fig. 7). As long as there was no microscopic connection between the submitted lymph node candidates and the gross description matched the counted number in a given block, we counted them as separate lymph nodes.

**Fig. 7:**
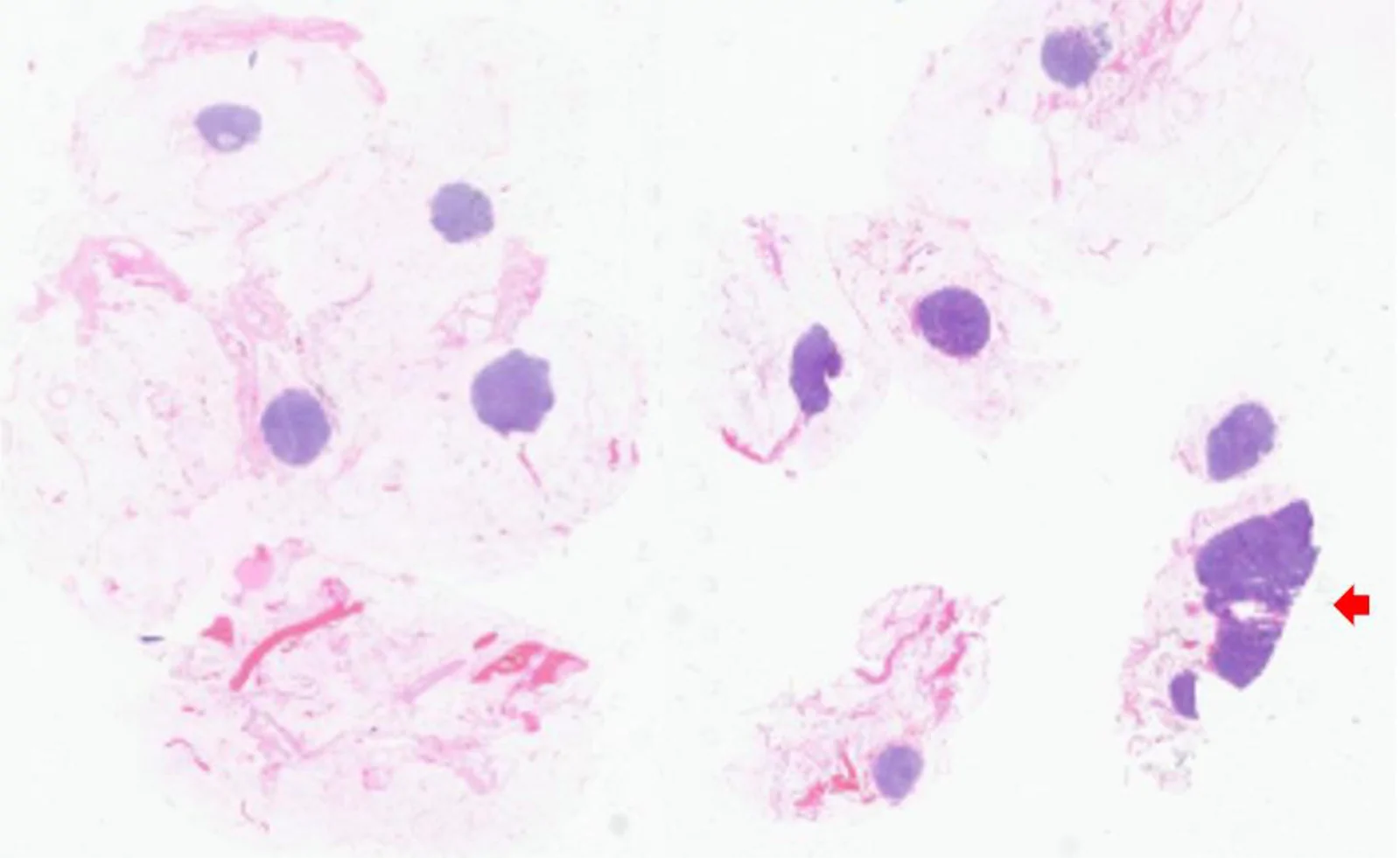
A collage of all the lymph nodes removed with the Cision InVision device in one case. Most lymph nodes appear intact; however, the bottom-right lymph node (red arrow) is most likely a fragment. These fragments are encountered in both conventional grossing and Cision InVision groups. (For interpretation of the references to color in this figure legend, the reader is referred to the web version of this article.)

It was considered whether awareness of subsequent device-based evaluation might have influenced the thoroughness of the gross examination (observer effect). To assess this possibility, we randomly pooled a case series before the introduction of the device to our department, and there was no significant difference between average lymph node numbers when compared to the study cases (*p* = 0.3), indicating that prosectors were not biased by the idea of an additional screening by a device.

Expectedly, the lymph nodes retrieved by the device were smaller than those found during the initial gross examination. These results were similar for positive lymph nodes as well. A trendline graph showed a positive correlation between lymph node size and the probability of positivity. We did not encounter any positive lymph nodes under 1 mm.

Ergonomically, the major improvable features of the Cision InVision device are the stage size and optical penetration depth of approximately 5–10 mm. With a larger stage and deeper optical penetration, the device might perform much better, and this would also reduce scanning time. Using the device adds to grossing time (average 30 min) when used as an additional step, which could create extra work for the gross room. Using the device also increases the submitted cassette count (4.2 per case); however, it prevents going back to specimens and submitting random fatty tissue.

Another perspective on a new device is undoubtedly the cost. We contacted the manufacturer to obtain current pricing and reimbursement context. At the time of writing, the listed price is approximately $65,000. An important factor that may materially influence the pricing of the technology is reimbursement coverage. SWIR examination of pathological specimens has been assigned to a Category III CPT add-on code (0961T) in conjunction with other CPT codes.

Jorns et al. previously evaluated the Cision InVision device and similarly demonstrated an increase in lymph node numbers.^10^ In that study, colorectal cases comprised 85 cases among 104 subjects. Among those, 34.1% of the 85 cases met the recommended minimum of 12 lymph nodes upon primary grossing. In contrast, in our study, all cases (100%) already met the 12-node requirement, with an average of 30 lymph nodes per case before device use. The observed additional increase in lymph node yield with device use in this context suggests that the Cision InVision device provides incremental value even when baseline grossing performance is already high, supporting its potential role as an adjunct rather than a replacement for standard gross examination.

This study serves as a proof-of-concept evaluation of the Cision InVision device, where we tried to answer the following two main questions: (1) Do we miss any lymph nodes in colorectal specimens despite high effort in lymph node search? (2) Do the missed lymph nodes pose any significance? Our study confirmed that despite the efforts of experienced prosectors, we are still missing lymph nodes. In two cases, the remaining fatty tissue had more positive lymph nodes. In one case, no positive lymph nodes were identified at initial grossing; however, one positive lymph node was found in the remaining fatty tissue, upstaging the case from node-negative to node-positive and potentially affecting treatment decisions and outcomes.

This study has several limitations. It represents a single-institution experience with a limited number of subjects, which may limit the generalizability of the findings. Another limitation is that the study did not include small specimens (under 10 cm). Although efforts were made to maintain consistency in grossing technique and personnel, minor variations in operator experience could have influenced lymph node yield.

In summary, in this “proof-of-concept” study with a limited number of subjects, implementing the Cision InVision device during colorectal specimen grossing appreciably increased lymph node yield, particularly by detecting smaller nodes that may be overlooked during conventional manual dissection. We observed additional positive lymph nodes in a subset of cases, resulting in upstaging in 2 out of 31 cases; more importantly, one case was upstaged from node-negative to node-positive disease. These findings may suggest that integrating optical imaging into routine gross examination may enhance the thoroughness and accuracy of nodal assessment in CRC. Future multi-institutional studies with larger cohorts are warranted to validate these results and to explore the potential of this technology in other specimen types and grossing environments.

## Ethics statement and patient consent

The Institutional Review Board (IRB) of Endeavor Health determined that the proposed activity is not research involving human subjects as defined by DHHS and FDA regulations. Therefore, IRB review and approval by this organization is not required in this study (IRB ID: IRB2025-0123).

## Declaration of competing interest

The authors declare that they have no known competing financial interests or personal relationships that could have appeared to influence the work reported in this article.
